# Dark Septate Endophytic Fungi Increase Green Manure-^15^N Recovery Efficiency, N Contents, and Micronutrients in Rice Grains

**DOI:** 10.3389/fpls.2018.00613

**Published:** 2018-05-04

**Authors:** Carlos Vergara, Karla E. C. Araujo, Segundo Urquiaga, Claudete Santa-Catarina, Nivaldo Schultz, Ednaldo da Silva Araújo, Fabiano de Carvalho Balieiro, Gustavo R. Xavier, Jerri É. Zilli

**Affiliations:** ^1^Departamento de Ciências do Solo, Universidade Federal Rural do Rio de Janeiro, Seropédica, Brazil; ^2^Departamento de Fitotecnia, Universidade Federal Rural do Rio de Janeiro, Seropédica, Brazil; ^3^Embrapa Agrobiologia, Seropédica, Brazil; ^4^Centro de Biociências e Biotecnologia, Universidade Estadual do Norte Fluminense Darcy Ribeiro, Campos dos Goytacazes, Brazil; ^5^Embrapa Solos, Rio de Janeiro, Brazil

**Keywords:** *Canavalia ensiformis* (L.), ^15^N, *Oryza Sativa* L., nickel, iron, DSE fungi, manganese, full grain

## Abstract

An understanding of the interaction between rice and dark septate endophytic (DSE) fungi, under green fertilization, may lead to sustainable agricultural practices. Nevertheless, this interaction is still poorly understood. Therefore, in this study, we aimed to evaluate the accumulation of macro- and micronutrients, dry matter, and protein and N recovery efficiency from *Canavalia ensiformis* (L.)-^15^N in rice inoculated with DSE fungi. An experiment under greenhouse conditions was conducted in a randomized complete block design comprising split-plots, with five replicates of rice plants potted in non-sterilized soil. Rice (Piauí variety) seedlings were inoculated with DSE fungi, A101 and A103, or left uninoculated (control) and transplanted into pots containing 12 kg of soil, which had previously been supplemented with dry, finely ground shoot biomass of *C. ensiformis* enriched with 2.15 atom % ^15^N. Two collections were performed in the experiment: one at 54 days after transplanting (DAT) and one at 130 DAT (at maturation). Growth indicators (at 54 DAT), grain yield, nutrient content, recovery efficiency, and the amount of N derived from *C. ensiformis* were quantified. At 54 DAT, the N content, chlorophyll content, and plant height of inoculated plants had increased significantly compared with the control, and these plants were more proficient in the use of N derived from *C. ensiformis*. At maturation, plants inoculated with A103 were distinguished by the recovery efficiency and amount of N derived from *C. ensiformis* and N content in the grain and shoot being equal to that in A101 inoculation and higher than that in the control, resulting in a higher accumulation of crude protein and dry matter in the full grain and panicle of DSE-rice interaction. In addition, Fe and Ni contents in the grains of rice inoculated with these fungi doubled with respect to the control, and in A103 inoculation, we observed Mn accumulation that was three times higher than in the other treatments. Our results suggest that the inoculation of rice with DSE fungi represents a strategy to improve green manure-N recovery, grain yield per plant, and grain quality in terms of micronutrients contents in cropping systems with a low N input.

## Introduction

Rice, along with corn and wheat, is the most important cereal in the world and is consumed by more than half of its population (Fao, 2016) for its supply of starch and proteins (Fitzgerald et al., 2009; Tian et al., 2009). However, rice production depends on the intensive use of fertilizers, particularly nitrogenous fertilizers, which generally have low recovery efficiency, with approximately half being subject to loss (Lassaletta et al., 2014), thereby generating environmental and economic problems (Chardon et al., 2012; Sutton et al., 2013). In contrast, the use of green fertilization combined with soil microorganisms, could be an economically positive strategy for rice production, reduction of nutrient losses, and improvement of soil fertility (Ambrosano et al., 2011; Cavagnaro et al., 2015).

DSE fungi, a diverse group of cosmopolitan endophytes, are conidial or sterile ascomycetes that generally have a brown to dark mycelium and melanized septate hyphae. They can colonize plant roots intercellularly and intracellularly, eventually forming microsclerotia, and can promote host plant growth without causing disease symptoms (Jumpponen and Trappe, 1998; Thormann et al., 1999; Jumpponen, 2001; Wilson et al., 2004; Diene et al., 2013; Knapp et al., 2015). These fungi colonize approximately 600 plant species of 320 genera and 114 families (Jumpponen and Trappe, 1998). In the *Poaceae* family, for example, DSE fungi isolated from the healthy roots of wild rice (*Oryza granulata* Nees et Arn. ex Watt. and *Oryza glumaepatula* Steud.) can colonize commercial rice [*Oryza sativa* (L.)] and promote its growth, without triggering any disease symptoms (Yuan et al., 2010; Pereira et al., 2011; Santos et al., 2017; Vergara et al., 2018).

The positive effects of inoculating plants with DSE fungi are more evident in plants supplied with organic sources of N and P than with inorganic sources (Newsham, 2011; Qin et al., 2017; Surono and Narisawa, 2017; Vergara et al., 2017). Organic compounds stimulate the saprophytic character of these fungi, which secrete a series of enzymes, such as endoglucanases, cellulases, amylases, pectinases, and laccases, and secondary metabolites that break complex compounds of carbon (C), nitrogen (N), and phosphorus (P), such as cellulose, starch, protein, and phytate, and convert these into nutrients for host plant absorption (Jumpponen and Trappe, 1998; Jumpponen et al., 1998; Caldwell et al., 2000; Choi et al., 2005; Usuki and Narisawa, 2007; Upson et al., 2009; Mandyam et al., 2010; Doolotkeldieva and Bobusheva, 2011; Berthelot et al., 2016; Adeoyo et al., 2017; Surono and Narisawa, 2017). Nevertheless, although the literature indicates that DSE fungi can help the host plant to acquire nutrients from organic sources of N, there are still few studies that have evaluated the growth and development of plants supplied with leguminous green manure as a single source of N.

In earlier studies, two isolates of DSE fungi, A101 and A103, isolated from wild rice (*O. glumaepatula*) were identified through ITS phylogeny as belonging to the order *Pleosporales* (suborder *Massarineae*) (A103) and to an unknown taxon (A101) (Ribeiro, 2011; Vergara et al., 2018). These fungi colonize wild (*O. glumaepatula*) and commercial (*O. sativa* [L.]) rice with no disease symptoms (Ribeiro, 2011; Vergara et al., 2018). A103 increases content of N and of other nutrients in rice plants cultivated under hydroponic conditions (Vergara et al., 2018), the fungus A101 increases the recovery efficiency of ^15^N, P and K and the content of macro and micronutrients of tomato plants fertilized with *C. ensifomis* (Vergara et al., 2017); both fungi increase growth of host plant. However, further studies are needed, to evaluate whether the positive response of host plant to inoculation with DSE fungi, observed under controlled conditions, reproduces under conditions of non-sterilized soil of greenhouse, as well as the effect of these fungi on plant development and crop yield.

Our hypotheses are that (i) the DSE fungi improve the recovery of N and other nutrients derived from the green manure *C. ensiformis* in rice plants and (ii) these fungi promote the growth of rice plants increasing the grain yield under condition of non-sterilized soil. The objectives of this study were to evaluate the accumulation of macro- and micronutrients, dry matter, and protein, and the efficiency of nitrogen (N) recovery from finely ground *Canavalia ensiformis* (L.)-^15^N in rice inoculated with dark septate fungi. To this end, we inoculated rice plants (Piauí variety) with the fungal isolates A101 and A103 and supplied these plants with the finely ground shoot biomass of *C. ensiformis* enriched with ^15^N. Under conditions of greenhouse soil cultivation, we determined the accumulation of nutrients (N, P, K, Ca, Mg, Fe, Mn, and Zn) and dry matter, the amount of N derived from green manure *C. ensiformis*-^15^N, and the recovery efficiency of N derived from green manure *C. ensiformis*-^15^N in rice plants at 54 (aboveground tissues) and at 130 days after transplanting (i.e., at plant maturation) (root, straw, and grain).

## Materials and methods

### Liming and fertilization of soil for experiment

The soil was sampled at 0–20 cm depth in an Integrated Agroecological Production System in Seropédica Municipality, RJ, Brazil. The soil was classified as Haplic Planosol (according to Brazilian Soil Taxonomy, or Planosol, under World Reference Base-FAO). The soil analysis showed the following chemical properties: pH = 5.47 in water; exchangeable Al^3+^ = 0.03 and H + Al = 1.86 cmol_c_ dm^−3^ (centimoles of charge per dm^3^ soil); Ca^+2^ = 1.21 and Mg^+2^ = 0.41 cmol_c_ dm^−3^; available P = 6.74, and K^+^ = 36.00 mg L^−1^; total *N* = 0.05% and C = 0.47%. The soil was classified as sandy soil (3% clay, 5% silt, and 92% sandy). Pots with 14 L capacity, corresponding to each experimental unit, were filled with 12 kg of a sieved and homogenized soil sample. Two months after lime addition (equivalent of 1.62 t ha^−1^; *MineralCal*) to correct for Ca^+2^ and Mg^+2^ deficiencies, the soil was fertilized with the equivalent of 27 kg P_2_O_5_ ha^−1^ (simple superphosphate), 13 kg K_2_O ha^−1^ (potassium sulfate), and 7 kg ha^−1^ micronutrient fertilizer as F.T.E BR-12 (fritted trace elements), according to Freire et al. (2013).

### Nitrogen fertilization with ^15^N-labeled green manure

For green manure fertilization, dry, finely ground shoot biomass of *C. ensiformis* [L.] was used. *C. ensiformis* (L.) is a legume widely used in tropical agriculture as a green manure for nutritional enrichment of soils (Rodrigues et al., 2004), contributing considerable amounts of N to the soil-plant system due to its association with fixing bacteria of N_2_ (Perin et al., 2003). *C. ensiformis* (L.) was prepared and applied according to Vergara et al. (2017). Plants of *C. ensiformis* were previously cultivated in ^15^N-enriched soil for use as green manure, and their dry aerial parts (dry 72 h at 65 °C) enriched with 2.15 atom % ^15^N (*C. ensiformis*-^15^N) was sampled around 60–70 days after germination (flowering period). The dry shoot of *C. ensiformis* was finely ground and then sterilized by gamma irradiation (25 kGy). The concentrations of macro (g kg^−1^) and micronutrient (mg kg^−1^) for *C. ensiformis*-^15^N were: *N* = 23.8; P = 2.0; K = 5.8; Ca = 12.3; Mg = 3.2; S = 1.9; Cu = 10.0; Fe = 792.0; Zn = 39.0; Mn = 50.0; B = 27.0; and C = 38.2% (Vergara et al., 2017). Each pot filled with 12 kg soil receiving 5.04 g of finely dry ground biomass of *C. ensiformis*, equivalent to 20 kg N ha^−1^, which was applied at one time and carefully homogenized in the soil before planting.

### Inoculum preparation and inoculation of the endophytes

The isolates of DSE fungi investigated here were isolated from *O. glumaepatula* and identified through the phylogeny of ITS (Ribeiro, 2011; Vergara et al., 2018). These fungi are maintained in the Centro de Recursos Biológicos Johanna Döbereiner (www.embrapa.br/agrobiologia/crb-jd) culture collection (A101 and A103). The ITS region sequences are maintained in GenBank (KR817246 = A101 and KR817248 = A103). The inoculum was prepared according to Andrade-Linares et al. (2011) and Vergara et al. (2017). Each isolate was grown in a 300 ml Erlenmeyer flask containing 150 ml of potato dextrose agar (PDA) medium for 2 weeks at 28°C under 80 rpm shaking. The fresh mycelium was filtered and washed with autoclaved distilled water until the liquid became clear to avoid carry-over of any material from the PDA medium into the inoculum. Then, the mycelium was weighed and part of it was mixed with autoclaved distilled water for 1 min at minimum speed using a mixer (Arno Optimix Plus, model LN27, Brazil) driving at laminar flow to avoid any kind of contamination. The viability of these fungi was checked by plating the suspension of the mycelium in the PDA medium, yielding 10^4^ colony-forming units. For inoculation, the suspensions were adjusted with autoclaved distilled water to a concentration of 1% (w/v).

### Experimental design, treatments, and growth conditions

The experiment with rice seedlings was conducted in a randomized complete block design comprising split-plots, under greenhouse conditions at Embrapa Agrobiologia, in Seropédica Municipality, RJ, Brazil. The experiment consisted of 30 plots: rice (*Oryza sativa* [L.] Piauí variety) plants inoculated with DSE fungi (isolates A101 and 103) or left uninoculated (control), two collections (54 and 130 days after transplanting) and five replicate blocks. Each plot was a pot with a 14 L capacity with one rice plant. Fifteen plots were collected at 54 DAT (i.e., vegetative state) and another 15 at 130 DAT (i.e., plant maturation), and each treatment had five replicate blocks. All treatments received *C. ensiformis*-^15^N as the sole N source. Piauí is a local landrace variety from the state of Maranhão-Brazil that is used in cropping systems with a low N input. Piauí has lower Michaelis-Menten constant (or high affinity) to nitrate uptake (Santos et al., 2011), especially when it is inoculated with a dark septate endophytic (DSE) fungus, A103 (Vergara et al., 2018) and higher nitrogen remobilization efficiency (Souza et al., 1998). Rice seeds were washed with 70% alcohol for 3 min and disinfected with 2.5% sodium hypochlorite for 3 min, followed by eight successive washes in autoclaved distilled water. subsequently, seeds were pre-germinated in water agar (8 g L^−1^) at 28°C in order to select homogenous plants for experiment (Vergara et al., 2017).

Rice seedlings were inoculated with DSE fungi by root dipping in the mycelial suspension (1% w/v) at 6 days after germination, while control plants only received autoclaved distilled water. The soil (12 kg) of inoculation treatments was also moistened by the 500 mL suspension (1% w/v) containing the inoculum, while the control pots only received autoclaved distilled water (Vergara et al., 2017). Pots were watered daily with 500 mL distilled water to maintain soil moisture around the field capacity (Vergara et al., 2017).

### Observations of disease symptoms and colonization

To examined whether the DSE fungi colonized the inner roots endophytically, the roots of rice plants inoculated with DSE fungi, A101 and A103, or left uninoculated were cleaned and fixed in 50% ethanol. After treatment with 2.5% potassium hydroxide overnight, roots were acidified with 1% hydrochloric acid overnight at room temperature and staining with 0.002% (w/v) methyl blue [a mixture of 10:9:1 glycerol/distilled water/hydrochloric acid (Phillips and Hayman, 1970; Koske and Gemma, 1989; Grace and Stribley, 1991)]. Root segments (~4 cm) were placed on slides with glycerin and hyphal structures were observed with an Axioplan light microscope (Carl Zeiss, Jena, 151 Germany) equipped with an Axiocam MRC5 digital camera (Carl Zeiss). Thirty root segments (McGonigle et al., 1990) were selected randomly for quantification of DSE colonization in each replicate block, and each treatment had five replicate blocks. In 100 microscopic fields, microsclerotia and intraradical hyphae were counted under 200 × magnification (Kohout et al., 2012). Disease symptoms were evaluated on scale of 0–3 (0: no visible symptoms; 1: light yellowing; 2: yellowing and late growth; 3: wilting or death) at 54 DAT (Diene et al., 2013; Mahmoud and Narisawa, 2013).

### Measurements

Stem diameter, plant height, tillers, and leaf number, total leaf area (LI-3100C area meter, LI-COR, Nebraska, USA), chlorophyll level (SPAD-502 meter, Konica-Minolta, Japan), shoot dry weight (65°C), concentration of N, P, K, Ca, Mg, Zn, Fe, Mn, and ^15^N abundance were determined in aboveground dry matter at 54 DAT. Root and straw dry weight, panicle weight, filled grain weight adjusted to 13% moisture and protein content were measure at 130 DAT (i.e., at plant maturation). Macronutrient concentration was determined in the root, straw and grain and micronutrient was only determined in the grain. Aboveground dry biomass (at 54 DAT), root, straw and grain (at 130 DAT) (dried at 65°C, 72 h) were crushed in a Wiley-type laboratory mill (<40 mesh) and their grain size were decreased by a rolling mill (Smith and Um, 1990). Micronutrient concentrations were determined in an aqua regia extract (ISO 12914, 2012) by a plasma detector (PerkinElmer® Optima™ 8300), while concentrations of macronutrient were obtained according to Tedesco (1982). The crude protein content in the grains was obtained by multiplying the grain N content by 5.95 (Juliano, 1985).

^15^N abundance was measured using continuous-flow isotope ratio mass spectrometry (Finnigan DeltaPlus mass spectrometer coupled to the output of a Carlo Erba EA 1108 total C and N analyzer—Finnigan MAT, Bremen, Germany) (Boddey et al., 1994). Contents of macro- (mg plant^−1^) and micronutrients (μg plant^−1^) were estimated as follow:

$$\text{Nutrient content }=\frac{\%\text{NC}\times \text{DM}}{100}$$

Where, NC is nutrient concentration (%) and DM is dry matter (mg plant^−1^ or μg plant^−1^).

After obtained the atoms % ^15^N excess by the difference between ^15^N abundance in plants and the ^15^N natural abundance in the air (0.3663% atoms), the fraction of ^15^N in the plant derived from finely ground *C. ensiformis*-^15^N (%fNdfGM), was calculated as describe by the International Atomic Energy Agency (IAEA, 2001) as follows:

%fNdfGM=100 ×(% N15 in excess in rice plant% N15 in excess in green manure )

With %fNdfGM value and the N content (mg plant^−1^) in plant material, it was possible to calculate the amount of N in the plants derived from *C. ensiformis*-^15^N (ANdfGM) as follows:

$$\text{ANdfGM }\left(\text{mg }{\text{plant}}^{-1}\right)=\frac{\%\text{fNdfGM}\times \text{Nitrogen content}}{100}$$

Finally, the recovery efficiency of ^15^N (%) by plants was calculate by the ANdfGM and the amount of applied N as ^15^N-labeled green manure (NGM) using to the following equation:

N15 recovery efficiency (%)=100                                            ×(ANdfGM (mg plant−1)NGM (mg pot−1))

### Statistical analysis

Data were submitted to analysis of variance (ANOVA), and the means were compared using *t*-test (LSD) (*p* < 0.05). ANOVA was performed after determining the normality of errors (Shapiro-Wilk) and the homogeneity of variance (Bartlett) of the data. The software R-project version R 3.4.1 (R Development Core Team, 2017) with the package ExpDes (Ferreira et al., 2013) was used for statistical analyses and data are presented as mean ± standard error.

## Results

### Observations of disease symptoms and colonization

The two isolates (A101 and A103) colonized the root tissue of rice plants abundantly with hyphae colonizing epidermis, cortex, and forming microsclerotia, with no disease symptoms. The fungi A101 and A103 formed 40 ± 0.6 and 33.3 ± 3.8% intraradical hyphae and 39.3 ± 0.9 and 46.7 ± 1.9% intraradical microsclerotia in roots of rice plants, leading to a total root colonization of 79.3 ± 0.9 and 80.0 ± 2.0%, respectively. Uninoculated plants (control) were poorly colonized by native DSE fungi with a total root colonization of 2%.

### Growth indicators and dry matter accumulation

Regarding growth and dry matter accumulation indicators, the DSE-rice interaction at 54 DAT showed a 16% increase in chlorophyll content and an increase in plant height (Table 1). However, no effect was observed in terms of the accumulation of aboveground dry biomass, stem diameter, numbers of leaves and tillers, or leaf area (Table 1). In contrast to plants at 54 DAT, at 130 DAT (i.e., at plant maturation) there was a significantly higher accumulation of dry matter of the full grain and panicle in the DSE-rice interaction compared with that of the control. In plants inoculated with A101, root dry matter accumulation was higher than that in the other treatments, although there were no differences between the treatments in terms of straw accumulation (Table 2).

**Table 1:** Growth indicators of rice plants (Piauí variety) at 54 days after transplanting (i.e., vegetative state). Plants were either uninoculated (control) or inoculated with dark septate endophytic fungi (A101 and A103) and fertilized with finely ground *Canavalia ensiformis* (L.)-^15^N as the sole organic N source.

**Treatment**	**Aboveground biomass (g plant^−1^)**	**Plant height (cm plant^−1^)**	**Stem diameter (mm plant^−1^)**	**Leaf number (unit plant^−1^)**	**Tiller number (unit plant^−1^)**	**Total leaf area (cm^2^ plant^−1^)**	**Chlorophyll level**
Control	4.5 ± 0.25	87.2 ± 1.36b	11.5 ± 0.45	18.20 ± 0.77	4.2 ± 0.20	439.2 ± 31.9	34.8 ± 1.58b
A101	4.5 ± 0.20	96.2 ± 3.06a	12.5 ± 0.23	18.75 ± 0.67	4.0 ± 0.32	394.5 ± 46.3	40.2 ± 0.61a
A103	4.7 ± 0.23	94.5 ± 1.24a	12.5 ± 0.17	19.75 ± 1.02	4.8 ± 0.37	445.0 ± 41.7	40.4 ± 0.48a
CV (%)	11.04	4.99	5.69	9.20	15.76	21.00	5.91

*Means ± SE (n = 5) followed by the same lowercase letter in the same column do not differ significantly, as determined by the t-test (p < 0.05). Absence of a letter indicates no significant difference, as determined by the F-test (p < 0.05). SE, standard error*.

**Table 2:** Root and straw dry weight, panicle weight, and filled grain dry weight with 13% moisture content at 130 DAT (i.e., plant maturation) of rice plants (Piauí variety) uninoculated (control) or inoculated with dark septate endophytic fungi (A101 and A103) and fertilized with *Canavalia ensiformis* (L.)-^15^N green manure as the sole organic N source.

**Treatment**	**Root dry weight (g plant^−1^)**	**Straw dry weight (g plant^−1^)**	**Panicle weight(g plant^−1^)**	**Filled grain dry weight (g plant^−1^)**	**Grain crude protein (g grain^−1^)**
A101	18.0, 0.7a	17.7, 0.9	10.7, 0.6a	7.51, 0.4a	0.79, 0.02ab
A103	13.9, 1.2b	16.3, 0.7	10.6, 0.6a	7.47, 0.4a	0.88, 0.03a
Control	14.3, 1.4b	16.6, 0.4	8.5, 0.3b	6.03, 0.3b	0.67, 0.02b
CV (%)	13.01	7.42	9.8	10.41	6.68

*Means ± SE (n = 5) followed by the same lowercase letter in the same column do not differ significantly, as determined by the t-test (p < 0.05). The absence of letters indicates no significant difference, as determined by the F-test (p < 0.05). SE, standard error*.

### Recovery efficiency of finely ground *C. ensiformis*-^15^N

**Figure 1:**
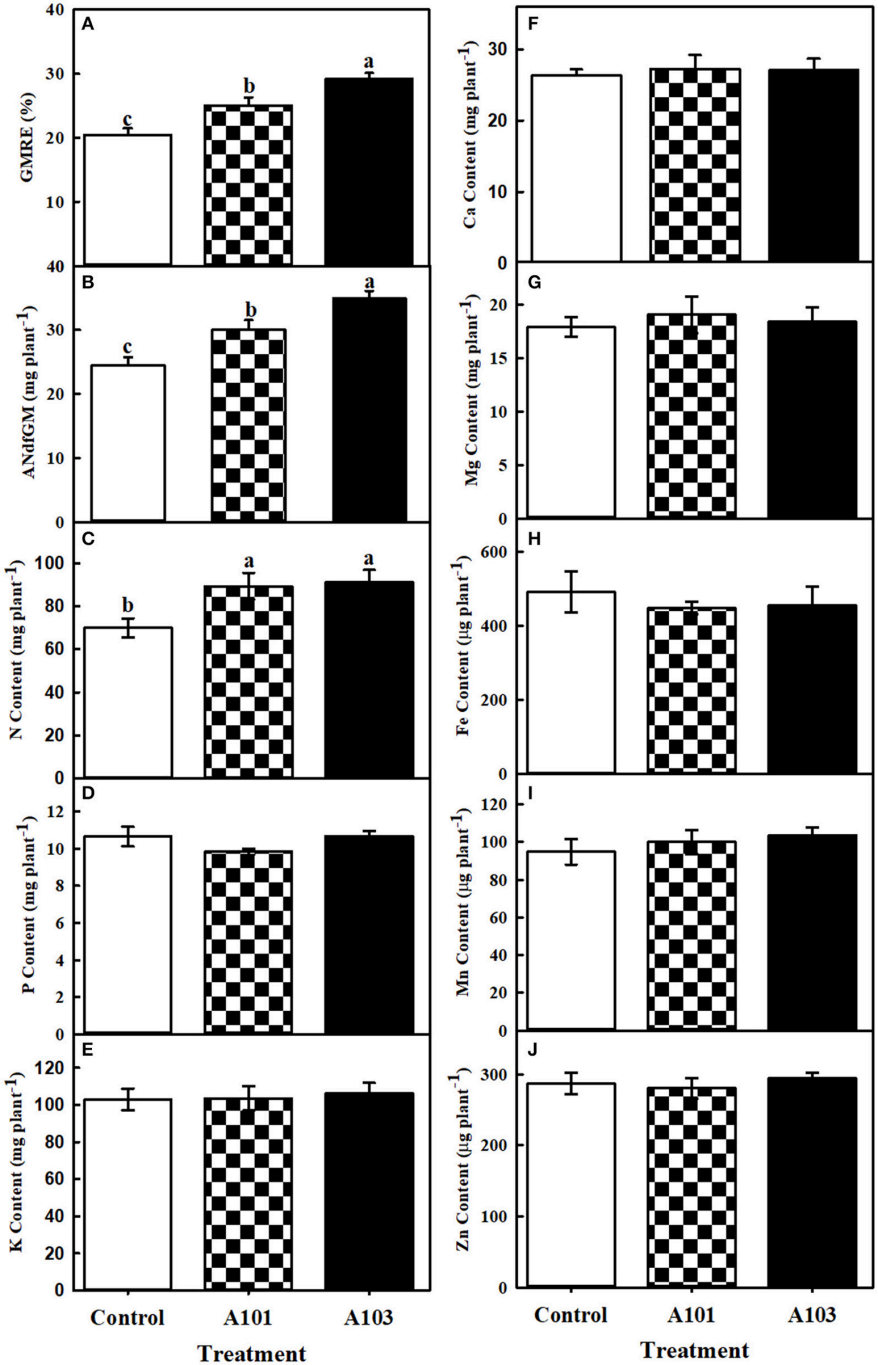
Recovery efficiency (GMRE) **(A)** and amount (ANdfGM) **(B)** of nitrogen derived from finely ground *Canavalia ensiformis* (L.)-^15^N; contents of N **(C)**, P **(D)**, K **(E)**, Ca **(F)**, Mg **(G)**, Fe **(H)**, Mn **(I)**, and Zn **(J)** at 54 days after transplanting of rice plants (Piauí variety). Plants were either uninoculated (control) or inoculated with dark septate endophytic fungi (A101 and A103) and fertilized with finely ground *Canavalia ensiformis* (L.)-^15^N as the sole organic N source. Among the treatments, values followed by the same lowercase letter do not differ significantly, as determined by the *t*-test (*p* < 0.05). The absence of letters indicates no significant difference by the *F*-test (*p* < 0.05). Error bars represent the standard error of the mean (*n* = 5).

After determining the abundance of ^15^N in dry matter, the recovery efficiency and amount of N derived from finely ground *C. ensiformis*-^15^N in the aboveground tissues (at 54 DAT) and in the root, straw, and grain (at 130 DAT) were determined in the treatments without (control) and with DSE fungi inoculation. At 54 DAT, the inoculated plants were more efficient in the recovery and use of N present in *C. ensiformis*-^15^N provided as the sole source of N, relative to the control, with significant increases of 23% for A101 and 43% for A103 in terms of the recovery efficiency and amount of nitrogen derived from *C. ensiformis*-^15^N in the aboveground tissues (Figures 1A,B).

**Figure 2:**
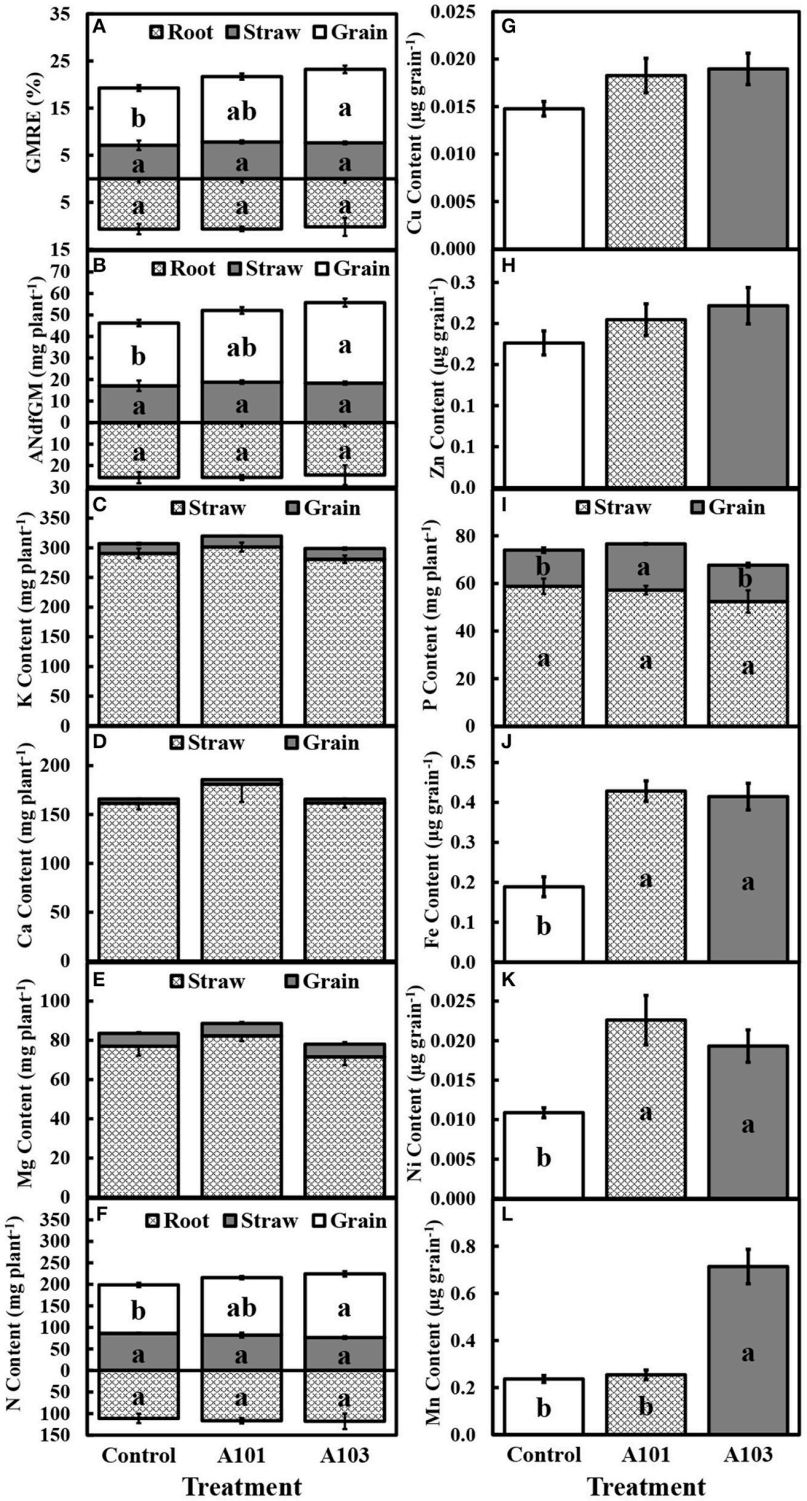
Recovery efficiency (GMRE) **(A)** and amount (ANdfGM) **(B)** of nitrogen derived from finely ground *Canavalia ensiformis* (L.)-^15^N; contents of K **(C)**, Ca **(D)**, Mg **(E)**, N **(F)**, Cu **(G)**, Zn **(H)**, P **(I)**, Fe **(J)**, Ni **(K)**, and Mn **(L)** at 130 DAT (i.e., at plant maturation) of rice plants (Piauí variety). Plants were either uninoculated (control) or inoculated with dark septate endophytic fungi (A101 and A103) and fertilized with finely ground *Canavalia ensiformis* (L.)-^15^N as the sole organic N source. Among the treatments, values followed by the same lowercase letter do not differ significantly, as determined by the *t*-test (*p* < 0.05). The absence of letters indicates no significant difference by the *F*-test (*p* < 0.05). Error bars represent the standard error of the mean (*n* = 5).

At 130 DAT, the recovery efficiency and amount of the N derived from *C. ensiformis*-^15^N in the grain, but not in straw and roots of rice inoculated with isolate A103 were comparable to those of plants inoculated with fungal isolate A101 and higher than those of the control treatment; however, there were no significant differences between these treatments in the recovery efficiency and amount of N derived from *C. ensiformis-*^15^N in the root and straw (Figures 2A,B).

### Nutrient accumulation

At 54 DAT, in the DSE-rice interaction there was a significantly higher accumulation of N in the aboveground tissues compared with the control (Figure 1C), which corroborates the observed higher recovery efficiency of N derived from *C. ensiformis*-^15^N (Figure 1A). Cu and Ni were not detected in the aboveground tissues at 54 DAT. The accumulations of P, K, Ca, Mg, Zn, Fe, and Mn were not affected by any of the inoculation treatments (Figures 1D–J). A similar effect was observed at 130 DAT for the accumulation of K, Ca, and Mg, in the straw, grain, and shoot (Figures 2C–E), as well as for the accumulation of N in root and straw (Figure 2F), and the accumulation of Cu and Zn in the grain (Figures 2G,H). In plants inoculated with the fungal isolate A103, the accumulation of N in the grain and in the shoot was equal to that in plants inoculated with fungal isolate A101 and greater than that of the control (Figure 2F), which corroborates the higher recovery efficiency of nitrogen observed in the grain and in the shoot of inoculated plants (Figure 2A). In plants inoculated with fungal isolate A101, there was a higher accumulation of P in the grain, which was significantly higher than that in the other treatments, although the same effect was not observed in the straw and shoot (Figure 2I). Similarly, in the grains of inoculated plants there was a significant accumulation of Fe and Ni, which was twice as high as that in the control (Figures 2J,K), and in plants inoculated with A103, Mn accumulation was three times higher than that of the other treatments (Figure 2L).

## Discussion

The two DSE fungi tested here were isolated from wild rice (*O. glumaepatula*) collected from the Amazon region and determined by the ITS phylogeny as belonging to the order *Pleosporales* (suborder *Massarineae*) (A103) and an unknown taxon (A101) (Ribeiro, 2011; Vergara et al., 2018). The intraradical melanized septate hyphae of these fungi, colonized epidermis and cortex cells of the roots of rice plants, and formed microsclerotia with no symptoms of disease as described by Vergara et al. (2018), Qin et al. (2017) and Yuan et al. (2010). Root colonization of rice plants by these two fungi was similar (~80%) in this study, in contrast to the findings of Vergara et al. (2018) under hydroponic conditions, where the colonization of roots by A101 and A103 isolates was 33 and 60%, respectively. Uninoculated plants (control) were poorly colonized by native DSE fungi with a total root colonization of 2%.

The cultivation of grasses (Newsham, 1999; Zijlstra et al., 2005; Upson et al., 2009; Qin et al., 2017) and other plant species (Usuki and Narisawa, 2007; Mahmoud and Narisawa, 2013; Surono and Narisawa, 2017) inoculated with DSE fungi and supplemented with organic sources of nutrients, under controlled conditions, suggest a higher nutrients recovery efficiency of inoculated plants than those that were not inoculated. Consistently, in the present study, we observed that inoculated plants, particularly those inoculated with fungal isolate A103, showed a more efficient use of N derived from a green manure of finely ground *C. ensiformis*-^15^N than control plants. There was an increase in the recovery efficiency and the amount of nitrogen derived from *C. ensiformis*-^15^N in the vegetative state (54 DAT) and at maturation (i.e., at 130 DAT), promoting a higher accumulation of N and chlorophyll content and greater plant height in the vegetative state. Furthermore, there was greater dry matter accumulation in panicles and grains and grain crude protein (Table 2) at maturation. These results indicate that inoculation with DSE fungi improves the utilization of N present in green manure in rice plants, although further studies are necessary to evaluate different *C. ensiformis*-^15^N doses and monitor plant responses to inoculation with DSE fungi during different periods of the growth cycle. In an earlier study, conducted under controlled hydroponic conditions, significant increases were also observed in shoot N content and dry matter of rice seedlings inoculated with DSE fungus A103, associated to a lower Michaelis-Menten constant (or high affinity) to nitrate uptake (Vergara et al., 2018).

The capability of DSE fungi to promote growth and the accumulation of N in rice plants supplied with only *C. ensiformis*-^15^N as an N source also suggests that these fungi can degrade organic compounds comprising C, N, and P and provide plant nutrients. In this regard, it has been stated that DSE fungi can degrade organic compounds such as cellulose, starch, protein, lipids, amino acids, gelatin, urea, and pectin under *in vitro* conditions (Caldwell et al., 2000; Menkis et al., 2004; Mandyam et al., 2010; Surono and Narisawa, 2017), and also promote the growth of grass (Newsham, 1999; Zijlstra et al., 2005; Upson et al., 2009; Qin et al., 2017) and other plant species, supplemented only with an organic N source or organic P source (phytate) under *in vitro* conditions (Usuki and Narisawa, 2007; Diene et al., 2013; Mahmoud and Narisawa, 2013; Surono and Narisawa, 2017). In addition, it has been shown that DSE fungi can produce proteolytic enzymes that degrade organic N compounds into N forms that are available to plants (Caldwell et al., 2000; Bizabani and Dames, 2016). For example, three isolates of *Phialocephala fortinii* have recently been shown to promote the growth of *Asparagus officinalis* (L.) in agar medium supplemented only with corn steep liquor (0.1%) or with corn steep liquor amended with inorganic nutrients (Surono and Narisawa, 2017). However, the mechanisms underlying increases in the contents of N and other nutrients and the accumulation of dry matter in plants inoculated with DSE fungi in relation to uninoculated controls are not yet fully understood. This fungus-plant association has been shown to be beneficial for the inoculated plants since the fungus colonizes the host plant and increases the contents of N and chlorophyll, plant height, and yield of rice grains, and does not cause the appearance of disease symptoms.

In addition to optimizing the use of N derived from *C. ensiformis*-^15^N and promoting a greater accumulation of dry matter, inoculation with DSE fungi A101 and A103 also increased micronutrient contents in rice grain. DSE fungi can also facilitate the uptake of micronutrients, such as iron, present in the soil (Bartholdy et al., 2001; Haselwandter, 2009; Vergara et al., 2017). In this study, in addition to inoculation with DSE fungi A101 and A103 doubling the Fe and Ni contents relative to the control, inoculation with A103 also tripled the Mn content of grain.

Rice plants preferentially accumulate more Mn than Fe (Mansfeldt, 2004), and can tolerate up to 5,000 mg kg^−1^ of Mn in the shoot without showing any symptoms of phytotoxicity, whereas other plants such as barley show symptoms of phytotoxicity at Mn concentrations of <150 mg kg^−1^ (Vlamis and Williams, 1964). Similarly, in this study, we observed that control plants and plants inoculated with A103 fungus accumulated more Mn than Fe.

The tripling of Mn content in plants inoculated with fungus A103 and the doubling of Fe content in those plants inoculated with both A101 and A103, compared with the control, suggests that inoculation with these fungi may increase the soil recovery efficiency of these nutrients or from fertilizer (in FTE BR12) and/or *C. ensiformis*-^15^N. This would optimize photosynthetic activity and other important processes for plant growth and development, which depend on Fe and Mn for correct functioning, contributing to higher grain quality and yield. Mn is essential for plants (McHargue, 1922) and is required in several processes, including photosynthesis, respiration, protein synthesis, hormonal activation, the activity of more than 30 enzymes, cell division, and root apex elongation (Burnell, 1988; Shao et al., 2017). In photosynthesis, for example, Mn catalyzes the photolysis reaction of water in photosystem II (PSII) (Schmidt et al., 2016). Similarly, Fe, which may limit the accumulation of grain dry matter in rice (*O. sativa*) (Takahashi et al., 2001), is also essential for plants, including the continued electron flow between PSII and photosystem I (PSI) (Eberhard et al., 2008; Briat et al., 2015), allowing photosynthetic CO_2_ fixation. The efficiency, structures, and functionality of the photosynthetic apparatus are all strongly dependent on Fe (Layer et al., 2010; Yadavalli et al., 2012). Fe is accordingly found in three of the largest complexes of the photosynthetic apparatus. Two Fe atoms are present in PSII and 12 atoms in PSI. Cytochrome *b*_6_*f* contains four Fe atoms and there are two atoms in Rieske-type proteins. In addition, the two complex antennae, the light-energy collectors associated with the two photosystems, contain chlorophyll, the synthesis of which is dependent on iron (Eberhard et al., 2008; Briat et al., 2015). This fact explains, in part, the higher levels of chlorophyll observed in vegetative plants inoculated with DSE fungi compared to the control.

Although details of the absorption and distribution of Fe and Mn following establishment of the DSE-rice interaction still remain obscure, the fact that inoculated plants have a tripled Mn content (in plants inoculated with fungus A103) and doubled Fe content in the grain suggests that these fungi may be potential siderophore producers, which would favor the absorption of these nutrients by inoculated plant. Indeed, the DSE *P. fortinii* synthesizes the siderophore hydroxamate and increases Fe (III) absorption in host plants (Bartholdy et al., 2001).

The increase in Ni content of grain observed in inoculated plants relative to the control also suggests a higher efficiency of recovery of this nutrient by inoculated rice plants. Although the Ni concentration required by plant species is very low (0.05–10 mg kg^−1^ dry mass) (Nieminen et al., 2007), this nutrient is also essential for plants (Eskew et al., 1983), being complementary to Mn and Fe by acting in other diverse metabolic processes, such as ureolysis and hydrogen metabolism. In this regard, Ni has been identified as a component of many enzymes, including urease, glyoxalases, peptide deformylase, methyl coenzyme M reductase, and a few superoxide dismutases and hydrogenases (Ermler et al., 1998; Küpper and Kroneck, 2007).

The quality of a rice grain is determined by the content and bioavailability of nutrients (Welch et al., 2005; Fan et al., 2008; Teklić et al., 2013; Briat et al., 2015) and by some post-harvest processing such as grain polishing (Jiang et al., 2008; Hansen et al., 2012; Briat et al., 2015)—a process that causes substantial losses of nutrients in the grains. An increase in the content and bioavailability of nutrients results in more vigorous plants (Briat et al., 2015), thereby increasing the yield of grains and their quality as staple food or as seeds (Juliano, 1993; Kranner and Colville, 2011; Briat et al., 2015). In the present study, the Fe, Zn, Cu, Ni, and Mn contents in rice grain of non-inoculated (control) and inoculated plants were within the range of values usually found in the literature (Chukwuma, 1995; Herawati et al., 1998; Gregorio et al., 2000; Wang et al., 2009; Teklić et al., 2013; Shraim, 2017). Additionally, symptoms of phytotoxicity were not observed during the experiment. These results indicate that inoculation of plants with dark septate fungi improved the quality of rice grains, especially in terms of micronutrients contents.

Hence, the results of the present study indicate that DSE fungi can help plants to acquire both macro- and micronutrients from complex substrates and consequently there is an improvement in physiological state, plant growth, and grain quality. An improvement in the grain quality would serve to combat micronutrient malnutrition, which affects more than three billion people worldwide, especially in the developing nations (Mahender et al., 2016).

## Conclusion

Rice plants inoculated with DSE fungi, particularly the fungal isolate A103, showed more efficient use of N derived from green manure *C. ensiformis*-^15^N, accumulating this element in both the vegetative state and in the full grain as crude protein. In addition, inoculation with these fungi increased the contents of Fe, Mn, and Ni in the grain, thereby improving grain quality and yield.

## Author contributions

CV, KA, SU, CS-C, NS, EdSA, FdCB, GX, and JZ: designed, performed experiments, and analyzed data. CV, KA, and JZ: conceived the experiments and wrote the paper. All authors read, edited, and approved the final manuscript.

### Conflict of interest statement

The authors declare that the research was conducted in the absence of any commercial or financial relationships that could be construed as a potential conflict of interest. The reviewer KHA and handling Editor declared their shared affiliation.
